# Selection of a classroom observation tool for disability inclusion in Uganda

**DOI:** 10.4102/ajod.v13i0.1485

**Published:** 2024-10-31

**Authors:** Elizabeth S. Kawesa, Claire Nimusiima, Janet Seeley, Femke Bannink Mbazzi

**Affiliations:** 1 Disability Research Group, MRC/UVRI & LSHTM Uganda Research Group, Entebbe, Uganda; 2 Department of Social Science, MRC/UVRI & LSHTM, Uganda Research Unit, Entebbe, Uganda; 3 Department of Global Health and Development, London School of Hygiene and Tropical Medicine, London, United Kingdom; 4 International Centre for Evidence in Disability, London School of Hygiene and Tropical Medicine, London, United Kingdom

**Keywords:** disability, adaptation, testing, classroom observations, tools, inclusive education, peer support, primary schools, Uganda

## Abstract

**Background:**

Obuntu Bulamu is a Ugandan intervention promoting inclusive education for children with disabilities. This culturally appropriate approach, based on the Ubuntu philosophy, utilises peer-to-peer support activities for children, parents and teachers.

**Objectives:**

To effectively measure the intervention’s impact on disability inclusion, the study aimed to select, adapt and test classroom observation instruments suitable for the Ugandan context.

**Method:**

Three structured classroom observation tools were selected and piloted in 10 primary schools in Wakiso District: The Classroom Observation Checklist (CoC), the Teacher-Pupil Observation Tool (T-POT) and the Interaction Engagement Scale (IES). These tools were adapted to ensure cultural relevance and applicability within Ugandan school settings.

**Results:**

Factors like class size, teaching methods, cultural relevance, language and ease of use influenced the suitability of the selected tool. The CoC emerged as a more effective tool with a strong internal consistency (Cronbach’s alpha of 0.80) for capturing inclusiveness and peer-to-peer support in the classroom compared to the T-POT and IES.

**Conclusion:**

The study findings emphasise the significance of adapting and testing tools in specific cultural contexts and low-income country settings and considering culturally contextual factors like class size, teaching methods, language complexity and ease of use when measuring disability inclusion in primary schools.

**Contribution:**

The selection of a classroom observation tool for the Obuntu Bulamu randomised control trial contributed to African disability knowledge and practices designed on and for the continent.

## Introduction

Inclusive education is a cornerstone of global development (UNICEF 2017), especially within the United Nations’ Sustainable Development Agenda, which emphasises the significance of removing barriers to learning and creating an environment where every child can participate and thrive (United Nations 2015). This inclusivity becomes particularly crucial in low-income countries (LIC) where children with disabilities often face exclusion or segregation from mainstream educational environments (Donohue & Bornman 2018; Montenegro & Valbuena 2009). Uganda has taken steps towards inclusive education as a proactive LIC, particularly through initiatives such as *Universal Primary Education (UPE) and the Disability Act* (Ministry of Education and Sports 2019). Despite these efforts, the effective measurement and implementation of inclusive practices, especially in large classroom settings common to LICs, remain challenging (Donohue & Bornman 2018). This article describes the selection, adaptation and testing of classroom observation instruments within the ‘Obuntu Bulamu’ study in Wakiso district, central Uganda, specifically the Classroom Observation Checklist (CoC), Teacher-Pupil Observation Tool (T-POT) and Interaction Engagement Scale (IES). Tools were selected, adapted and tested to provide the study with culturally sensitive and effective means of measuring disability inclusion in primary schools. Through systematic observations, this research aims to offer insights into the selection, adaptation and testing of tools that could guide inclusive practices tailored to LIC like Uganda.

## Background

### Inclusive education

The United Nations Convention on the Rights of Persons with Disabilities (CRPD) articulates disability as a long-term physical, mental and intellectual impairment that limits one’s functioning, full and equal participation (United Nations General Assembly 2006). The United Nations defines inclusive education as providing equal access to quality education for all children, regardless of their background, aptitude or special needs (UNICEF 2017). The right to inclusive education is highlighted by the Convention on the Rights of Persons with Disabilities (United Nations General Assembly 2006). Goal 4 of the United Nations’ Sustainable Development Agenda is to guarantee access to quality, lifelong education for all (United Nations 2019). Inclusive education is precious because of its promotion of mutual growth and learning among students from different backgrounds (UNICEF 2020); however, in LIC, many children with disabilities do not go to school and are faced with various barriers, poor access and a lack of adequate facilities (World Health Organization and United Nations Children’s Fund 2023). While several African countries are on board to improve inclusion and have signed the CRPD, the implementation of policies is still low (Mendoza & Heymann 2024; Oyaro 2015). The 2024 progress report shows a decline in access and inclusion in education for all, because of a lack of resources and an imbalance pupil-teacher ratio (Sachs, Lafortune & Fuller 2024). A systematic review exploring inclusive education interventions (Mendoza & Heymann 2024) only found 31 studies in LICs, highlighting the need for more culturally appropriate interventions to boast inclusive education.

Uganda, hailed as one of the most engaged LICs in adopting inclusive education (Abimanyi-ochom & Mannan 2014), started with the introduction of Universal Primary Education (UPE) and later Universal Secondary Education (USE) (Ministry of Education and Sports 2019). With the introduction of UPE, the number of children with disability enrolling in school increased (Kan & Klasen 2021; Ministry of Education and Sports 2017). However, this surge in numbers did come with challenges (Kan & Klasen 2021). In a qualitative study to understand the implementation of UPE schools, Kyambadde and Khumalo (2022) found misappropriation of funds, low stakeholder involvement and other factors hindering the success of this programme. Children with disabilities are more affected and less included, even with such programmes, as parents are still tasked with payments and yet many are from very poor backgrounds (Lamichhane & Tsujimoto 2023). Collaborative approaches with government organisations, other organisations and stakeholders like parents and community members are critical for inclusive education implementation (Mendoza & Heymann 2024).

A study conducted in Finland and South Africa (Engelbrecht et al. 2017) found that inclusive education cannot be implemented in the same manner across different countries because of contextual differences. In high-income countries (HIC), the classrooms are relatively small, with 25–30 students per class (Cameron 2014), while the majority of schools in LICs face challenges with implementation because of large class sizes, poor disability attitudes, lack of collaborative approaches with parents and rigid teaching strategies among others (Mitchell & Sutherland 2020). Inclusive education methods vary from school to school and might include setting targets for learners, one-on-one support and provision of support (Schuelka & Johnstone 2012). The strategy of children working together in regular classes (Schuelka & Johnstone 2012) and working in pairs or small groups (Mitchell 2017) as ‘peers’ has been highlighted as an important contributor to inclusive education (Banda, Hart & Liu-Gitz 2010; Wang et al. 2015). A study of a peer-mediated intervention among children on the autism spectrum determined that when children received and provided support, they benefitted from peer-to-peer relationships (Carter et al. 2017). The approach also allows teachers to support large classrooms, as peers can support each other during play and other activities (Mccurdy 2014). Peer-to-peer support is a promising inclusion method for LICs because of the higher teacher-pupil ratio and limited access to resources and teaching materials (Ainscow & Miles 2008; Bannink Mbazzi et al. 2020).

### ‘Obuntu bulamu’ – The conceptual framework

This classroom observation study was nested within the Obuntu bulamu peer-to-peer support project, an African research study to improve the inclusion and participation of children with disabilities through an intervention rooted in the Ubuntu philosophy (Bannink Mbazzi et al. 2020; Nimusiima et al. 2024). ‘Obuntu bulamu’ is the Luganda word for Ubuntu, used in Central Uganda, which describes the philosophy of humanity and reciprocity (Murove 2012; Owusu-Ansah & Mji 2013).

The Obuntu bulamu intervention (Bannink Mbazzi et al. 2020) was designed by children with disabilities, their peers, parents, teachers, community leaders, academics, and health and rehabilitation workers. After the co-creative development, the intervention was pilot-tested for feasibility over two academic years, in which educators, parents and students participated in a series of training and support sessions designed to improve the participation and inclusion of children with disabilities in school (Bannink Mbazzi et al. 2020). The intervention aims to enhance social responsibility by using culturally appropriate training and support methods and local resources (Bannink Mbazzi et al. 2020). It is expected to lead to better education access, retention and outcomes, inclusive interactions in schools, greater participation in daily home activities, improved community attitudes and involvement and active participation of the children and their families in research ultimately enhancing their quality of life. This is a significant and central element in the African disability discourse (Berghs 2017; Mutanga 2023) which aligns with the sustainable development goal (SDG) agenda to promote inclusion and disability discourse.

### Measuring inclusive education in classroom settings

Compared to self-report ratings, classroom observations have been identified as a more reliable indicator of peer and teacher interactions and inclusive practices in classrooms in HIC (Hora & Ferrare 2013; Martin et al. 2010; OFSTED 2018). Several classroom observation instruments have been created and deployed for use in HICs (Martin et al. 2010). The Inclusive Classroom Profile (Soukakou, Evangelou & Holbrooke 2018), the T-POT (Martin et al. 2010) and the Stallings Classroom Observation Systems (Stallings, Knight & Markham 2014) are just a few examples.

Inclusive education studies measuring interaction and classroom inclusion in sub-Saharan Africa have primarily focussed on teachers and parents’ perceptions with data collected through surveys and interviews (Carew et al. 2018; Engelbrecht et al. 2017; Richards & Farrell 2012; Wong et al. 2015). For instance, a study on the barriers to inclusion in Kenya and Uganda found that parent and community attitudes, large class sizes, limited teacher resources and inadequate training are significant obstacles to inclusive education (Donohue & Bornman 2018; Ngwaru 2015). However, few studies have systematically observed classroom interactions (i.e. peer and teacher-pupil interactions) to determine the inclusiveness of educational practices (Blatchford 2003). This study addresses this gap by selecting, adapting and testing tools that are culturally relevant and feasible to use in Ugandan primary schools.

In classrooms in LICs, barriers such as large class sizes, limited resources and insufficient teacher training often limit the effectiveness of inclusive education practices (Donohue & Bornman 2018; Okkolin, Lehtomäki & Bhalalusesa 2010). For example, some studies have shown that students with disabilities receive less attention and have fewer interactions with their teachers and peers than their peers without disabilities (Ngwaru 2015; Okkolin et al. 2010). Studies have found evidence that teachers often lack the skills or knowledge to manage or integrate children with disabilities into their classes (Mitchell & Sutherland 2020), leaving them excluded from mainstream classrooms.

A few studies have collected data through classroom observations in sub-Saharan Africa (Filmer, Molina & Stacy 2015; Salzano and Labate, 2016). The classroom observations used noted components from distinctive survey data sets (Filmer et al. 2015), classroom-based assessments (Salzano and Labate, 2016), free flow and controlled interviews and taking of notes (Engelbrecht et al. 2015). Engelbrecht et al. (2015) used classroom observations to document the happenings of a typical school day and found that using separate classrooms for children with disabilities only enforced stereotypes and did not help the students feel included or enhance peer-to-peer support. Observing interaction between peers, children and teachers is feasible using available classroom observation tools (Martin et al. 2010). These measures help to understand the extent of inclusion, and whether the child with disability is included and integrated into the classroom (Singal 2008). Adaptations are required to use the same tools in the LIC, as classrooms vary from 60 to 100 students per teacher and have a different set-up and availability of materials compared to most schools in the HIC (Kristensen, Omagor-Loican & Onen 2003). Understanding and applying methods and practices rooted in our own cultural settings is crucial for the development and transfer of knowledge (Mkabela 2005; Mutanga 2023).

There is limited research on using standardised classroom observation tools (CoC) as educational evaluation measures in Uganda. To the best of our knowledge, only the CoC has been used in a pilot study conducted in Uganda (Bannink, Idro and Van Hove, 2016) and in research conducted in schools within Zambia and Tanzania (Miles 2011). We did not find any published research on the use of other classroom observation tools in sub-Saharan Africa.

### Systematic observation framework

This article describes the testing of classroom observation tools used to measure disability inclusion within the ‘Obuntu Bulamu’ peer-to-peer support intervention study in Wakiso district, central Uganda. We used the systematic observations framework (McCall 1984) which provides a foundation for designing, conducting and interpreting observations (Van der Mars, Timken & McNamee 2018). This framework aids in identifying or developing observation tools that are both valid and reliable, enhancing the accuracy of the collected data.

In the context of our study on disability inclusion in Ugandan primary schools, the systematic observation theoretical framework informed a structured and standardised approach to observing and collecting data on classroom behaviours. Additionally, the framework enabled establishing clear criteria for selecting observation tools, aligned with research objectives and the specific needs, in our case studying peer-to-peer support and inclusion in Ugandan classrooms. The framework ensured that the selected tools were applied consistently across different observations and observers, thus enhancing the reliability and comparability of the data collected.

## Research methods and design

### Study design

The ‘Obuntu bulamu’ peer-to-peer support intervention followed a mixed-method Afrocentric study design conducted in 10 schools in the Wakiso district in Uganda. This study utilised a combination of culturally adapted ‘international standards and tools’ which align with the challenge of conducting research from an indigenous perspective (Chilisa 2017). The children observed in this study were part of the feasibility study that tested the ‘Obuntu bulamu’ intervention in 10 elementary schools in Wakiso, Central Uganda between 2017 and 2019 which included a total of 64 parents (33 parents of children with disabilities and 31 parents of the peers). Utilising a stratified random sampling approach, five private and five public schools were selected, with a focus on geographic distribution – two private and three public schools in semi-urban areas, while the rest (five) were in urban areas.

### Study population

This study encompassed 80 classroom observations, tracking 32 children with disabilities (14 boys and 18 girls) and their peers across baseline, midline and end-line assessments. Two children with disabilities could not be observed on several occasions because of absenteeism.

Participants comprised 32 children with disabilities, aged 8–14, selected through purposive sampling to ensure diversity in disability type, gender, age and socioeconomic status. Each child with a disability identified a peer for peer-to-peer support, resulting in a cohort of 64 participants (32 children with disabilities and 32 peers), each pair accompanied by at least one parent and one educator. Teachers and heads of schools supported the research team in screening students in their schools with disabilities for eligibility. The *Ugandan disability definition of the Disability Act* (in line with the CRPD) was used to define impairment (Uganda Parliament 2019). Schools informed parents of children in the school about the programme and screening process. The research team selected index children taking into account the different demographic attributes mentioned in Table 1. After screening and pre-selection through the school lists, parents were asked to consent. After obtaining consent from parents and, assent from children with disabilities, we proceeded with screening their peers. The children were asked to identify their peers, choosing a friend in class that supported them. Teachers supported in pre-screening peers, especially for the children with severe disabilities by selecting peers they had observed being supportive and friendly to the child with disabilities during class and break time. When the peers expressed their interest in the child and intervention, their parents were also contacted to discuss the study information and obtain consent. Where possible children with disabilities and their peers assented.

**TABLE 1:** Socio-demographic and class data characteristics of study participants (children and parents).

Variable	Level	Children with disability (*N* = 32)				Peers (*N* = 32)			
		Mean (s.d.)	Min–Max	*n*	%	Mean (s.d.)	Min–Max	*n*	%
Age-child	-	10.5 (2.05)	-	-	-	9.34 (1.84)	-	-	-
	-	-	08–15	-	-	-	07–14	-	-
Class-child	Nursery school	-	-	5	15.63	-	-	4	12.50
	P.1 to P.3	-	-	19	59.38	-	-	20	62.50
	P.4 to P.6	-	-	8	34.40	-	-	8	34.40
Household size	-	4 (2)	-	-	-	6 (4)	-	-	-
	-	-	0–9	-	-	-	0–20	-	-
Gender-child	Female	-	-	18	51.43	-	-	17	48.57
	Male	-	-	14	48.28	-	-	15	51.72
Parents with all household children attending school	-	-	-	22	59.50	-	-	15	40.50
Child’s mobility	Walking	-	-	26	81.82	-	-	32	100.00
	Crawling	-	-	1	3.03	-	-	0	-
	Assistive devices	-	-	5	15.00	-	-	0	-
Child’s way of communication	Non-verbal gestures	-	-	3	12.12	-	-	1/32	3.23
	Verbal speech 1–2-word phrases	-	-	9	27.27	-	-	2/32	6.45
	Verbal speech full sentence	-	-	20	60.61	-	-	28/32	90.32
Impairment – child (*as defined by parent and teacher following Uganda’s disability definition and verified with medical records where available*)	Autistic spectrum disorder	-	-	4	12.50	-	-	0	-
	Down syndrome	-	-	2	6.25	-	-	0	-
	Hearing impairment	-	-	4	12.50	-	-	1	20.00
	Hydrocephalus	-	-	4	12.50	-	-	0	-
	Intellectual disability	-	-	8	25.00	-	-	0	-
	Muscular dystrophy	-	-	2	6.25	-	-	0	-
	Physical disability	-	-	1	3.13	-	-	3	60.00
	Spinal bifida	-	-	4	12.50	-	-	0	-
	Visual impairment	-	-	3	9.38	-	-	1	20.00
Use of assistive devices-child	Walking	-	-	21	77.78	-	-	12	100.00
	Crawling	-	-	1	3.70	-	-	0	-
	Assistive devices	-	-	5	18.52	-	-	0	-

*Source:* Nimusiima, C. et al., 2024, ‘Adaptation and validation of the Child and Family Follow-up Survey (CFFS) tool to measure participation of children with disabilities in Uganda’, *African Journal of Social Work* 14(1), 20–30.

s.d., standard deviation.

Table 1 describes the social demographic, impairment and education characteristics of the children and parents.

The average age of the children with disabilities was 10 years, while their peers averaged 9 years. The majority of the children could walk independently while 18.5% used assistive devices. More than half of the participants (59.4%) were in primary years 1–3, with 2/3 of the children having a neurodevelopmental (autism spectrum disorder, Down syndrome and intellectual disability) or neurological (spina bifida and hydrocephalus) impairment.

### Classroom observation tools

Based on a scoping literature review including the words ‘classroom observation scale (or tool or list)’ and ‘disability’ (or impairment or special needs) in Google Scholar in 2018, the following classroom observation tools were found and reviewed: the Stallings Classroom Observation Systems (Stallings et al. 2014), the Classroom Observation Tool (Van Tassel-Baska et al. 2003), the CoC (Collins 2012), the T-POT (Martin et al. 2010) and the Interaction Engagement Scale (Hunt et al. 1996). Based on initial reviews by two of the researchers, three checklists were selected as most appropriate for our setting and study purpose: The CoC, T-POT and IES. Selection was based on the usage of the tools with children with disabilities, cultural appropriateness of observation items to our setting, number of observers required and duration of observations for feasibility (maximum 2 observers and not more than 1 hour per observation).

The *Classroom Observation Checklist* (CoC) is a tool designed to assess classroom interactions based on the principles of inclusive education. It is designed to assess various aspects of a child’s interaction and engagement in an inclusive classroom setting. The checklist (Collins 2012) is a resource from the index of inclusion (Booth et al. 2002). The index of inclusion is a comprehensive resource developed for improving inclusion, equity and participation in schools (Booth et al. 2002) and allows users to tailor the resources to their contexts for enhancing inclusion (Engelbrecht, Oswald & Forlin 2006). The index has multiple features to it and one of the significant features is questionnaires. The checklist is a practical tool that emerged from the index of inclusion and helps educators observe and reflect on the inclusive practices of a classroom environment (Collins 2012). It was piloted in Uganda in a study exploring the accessibility and inclusion of children with spina bifida in primary schools (Bannink, Idro and Van Hove, 2016). This checklist evaluates inclusive practices involving the index child, peers and teachers. The index of inclusion has been used in several studies (Duke 2009) and is well-documented for the promotion of inclusive education (Hick* 2005). The checklist comprises two items that focus on various aspects of inclusion, such as interaction quality, teaching strategies and classroom dynamics. The observation period for each session, typically core subjects like English, Mathematics or Social Science, lasts 20 min. Observers rate each item as ‘agree’, ‘agree to some extent’ or ‘disagree’, and not observed with space provided for additional notes to contextualise each observation.

The *Interaction and Engagement Scale* (IES), developed by Hunt et al. (1996) and widely used in the United States of America, is designed to assess classroom interaction and engagement. Observations are conducted in 10-min intervals, segmented into 20 intervals. During each segment, the observer documents the index child’s interaction type, participant involvement and interaction nature (e.g. request with a teacher). The scale also captures interaction quality (positive, neutral and negative), engagement levels (active, passive and not engaged) and grouping patterns (individual or group), providing a comprehensive view of classroom dynamics.

The *Teacher-pupil Observation Tool* (T-POT) measures behaviours and interactions between teachers and pupils within the classroom. The T-POT was integrated from other studies and developed to measure teachers’ interaction, index child and peers (Martin et al. 2010) and has been refined and adapted in several studies (Martin-Forbes, 2009; Gallucci, 2014). It has been used in various regions, including Gwynedd, North Wales and Ireland (Martin et al. 2010). It was designed to systematically record and assess both the child’s and teacher’s behaviours within a classroom setting. The T-POT is organised into two main sections: child–child and teacher–child interactions and behaviours. The tool includes specific behaviour indices, accompanying notes for detailed observations and an observation manual for the coder or observer to use as a guide. The tool uses a combination of tallies and notes to capture the frequency and context of the observed behaviours.

In the Child Behaviour section of the T-POT, the observer systematically records instances of aggression towards peers, noting whether the aggression was verbal, physical, destructive or disruptive. If no aggression is observed, the observer marks this as ‘Not observed’. The section also includes a component for tracking peer interactions initiated by the child or directed towards the child, such as ‘I-P’ (Initiation by Peer), ‘P-I’ (Peer Initiation) or ‘C-I’ (Child Initiation). The observer further documents the child’s response to these interactions, categorising them as Positive, Negative or Neutral. Additionally, the observer notes the percentage of time the child is off-task, such as ‘On task, 80%’, indicating the child’s level of focus during the observation period.

The Teacher Behaviour section of the T-POT is focussed on capturing the teacher’s responses to classroom dynamics. The observer records instances where the teacher ignores specific behaviours, particularly when the child is aggressive towards the teacher. Additionally, the observer tallies each time the teacher asks a question and tracks the child’s compliance or non-compliance with these questions. Detailed notes can be added to provide context on the child’s responses or attempts at compliance. This section also monitors the teacher’s use of indirect and direct commands, documenting whether the child had the opportunity to comply (‘No Opp’ for No Opportunity) and whether compliance or non-compliance occurred. Tallies are used for each instance, and notes provide further insights into these interactions. The section also includes space to document the use of time-out warnings, recording whether the child complied, did not comply or had no opportunity to comply.

A background information sheet was designed by the research team to capture relevant contextual details that may influence the observations such as subject, lesson duration and class size. The observation documents were printed separating assessments of teacher and child behaviours with dedicated note sections. The observation period for all observations was set to 20 min per child.

### Data collection

Two Ugandan graduate research assistants, specialised in clinical and educational psychology with prior experience in Ugandan classroom observations, conducted the data collection and classroom assessments. The two observers who had met the teachers, parents and children during the consenting process, were introduced as visitors and often sat in the front or back of the classroom depending on the layout and arrangement of the classroom and sitting arrangement of the children to be observed. Twenty minutes were used for the independent observation of the index child, peer, teacher and general classroom interactions. Each index child and peer was observed using the three selected tools separately.

The three tools were tested in 10 schools. The research team documented various classroom parameters such as teaching strategies, class size, subjects taught, seating arrangements and classroom divisions into pairs or groups. Each child, along with a peer, was observed thrice: at initial and subsequent years of testing the Obuntu Bulamu peer-to-peer support intervention. At different stages – baseline (pre-intervention), midline (during intervention) and end line (post-intervention) – the observers evaluated the usability, relevance and applicability of three assessment tools: the CoC, the Interaction and Engagement Scale and T-POT.

During baseline, the observers focussed on using the tools in their original format and observing the classroom setting, interactions within the classrooms and how well the items of the tools were observable and reflective of the classroom context.

All observations began with the identification of the index child, their peer and observer initials and noting other critical background information such as the class, number of children in the class and subject being taught, methods used during the observation, seating arrangement, specific data and time of the observation, plus the duration of the observation. The CoC, T-POT and IES were manually completed for each child with a disability and their peer.

When using the CoC, the observers evaluated various statements related to the inclusive practices within the classroom assessing the child’s participation, social interactions and the teachers’ support, the child’s engagement and behaviour and the overall classroom environment. Each statement was marked as either ‘Agree, Agree to some extent, Disagree, Not Observable and Not Applicable’.

During the IES use, observers conducted observations in 15 s intervals as per IES guidelines. During each interval, they captured the first communicative interaction involving the index child, noting the interaction’s function, quality and engagement level.

During the T-POT use, observers recorded observed instances with tallies; for example, if a teacher asked a question, this would be tallied and the child’s compliance would also be tallied. Notes sections were provided for each instance where the observers would provide further insights into these interactions.

All observation data were entered into Open Data Kit which was used as the data management tool for the study. The study team was trained on data entry and quality control using this tool. Subsequently, tablet computers facilitated the transfer of this information into the Open Data Kit (ODK) system, ensuring secure storage in locked computers and cabinets throughout and after the data collection phase.

### Data analysis

Data cleaning and analysis were performed using STATA post-entry. T-tests were used to compare the observational data across different phases of the study to assess the effectiveness of each tool (CoC, IES and T-POT) in capturing changes because of the intervention. Demographic and impairment information were analysed using frequency counts to gain insights into participant characteristics. Cronbach’s alpha coefficient was calculated to measure the internal consistency (reliability) of each observation tool and content validity to identify if the items in each tool adequately covered the observed behaviours in the classroom contexts.

To ensure reliability, the IES includes procedures for calculating inter-observer agreements. Differences between observers’ recordings were noted, tallied and analysed to determine the level of agreement. The T-POT tally counts were recorded for each behaviour and frequency run.

### Ethical considerations

Ethical clearance to conduct this study was obtained from the Uganda Virus Research Institute, Research Ethics Committee (No. GC/127/18/02/633), the Uganda National Council of Science and Technology (No. SS 4557) and the Ethics Committee of the Faculty of Psychology and Educational Sciences of Ghent University (No. Amendment 2017/06/Femke Bannink Mbazzi). All adults gave written informed consent to participate in the study. Parents of children and peers who were observed had consented using written consent following ethical approval of the ethics committee. Where possible, children had assented. Heads of schools and teachers too orally consented to participate in the study and be observed in their classrooms. Overall permission to conduct the research was obtained from the Uganda National Council for Science and Technology (SS 4557).

All procedures performed in studies involving human participants were in accordance with the ethical standards of the institutional and/or national research committee and with the 1964 Helsinki Declaration and its later amendments or comparable ethical standards.

## Results

### Adaptations of the tools

Using the systematic observations’ theoretical framework, which emphasises the importance of designing, conducting and interpreting observations in a structured and reliable manner, the research team discussed baseline findings and proposed changes to ensure the tools reflected the classroom setting, teaching strategies, cultural relevance, language and ease to use.

The baseline phase allowed the observers to identify items in the tools that were either not easy to capture or did not apply to the classroom contexts. Observations and suggested changes were discussed with the research team and in consultation with teacher representatives in the study team, the following changes were made.

In the CoC, the team replaced the following items: item 5, ‘the display of child’s work’ was replaced with ‘praising of achievement by teacher’ as children’s work is usually not displayed in Ugandan classrooms; item 17 ‘understanding homework’ was replaced by the ‘teacher explaining the homework to the child’ as we could not measure understanding during observations; and item 19, ‘family’s impression of the school’ was replaced with an overall item of ‘classroom being inclusive of children with disabilities’ as the families’ impressions could not be observed.

The IES had a structure that could be followed for each individual child and their peer; therefore, there were no changes made to the IES and the original items were used in all observations.

In adapting the T-POT for this study context, several key modifications were made for enhanced measurability and relevance. The tool was refined to separately assess teacher and child behaviours, with dedicated note sections for each component, allowing focussed observations. This included recording the observed interactions on a separate page. Initially, the original form had the teacher and child behaviours on one page. We also extended the observation period to 20 min using a single sheet unlike the original T-POT, which utilised the 5-min observation intervals per sheet. During the baseline observations, one sheet was used every 5 min which was not suitable in the context as 5 min could go by without observing any interaction in the entire class, as large parts of the lessons in our setting consist of teachers instructing and children looking at the teacher or copying notes from the blackboard. In addition, observers noted that the shorter intervals in the original tool often led to a heightened focus on timekeeping and recording, detracting from the overall classroom observation.

These adaptations ensured that the tools were culturally sensitive, feasible, easy to use and time efficient, adhering to the principles of systematic observation.

### Evaluation criteria

For all classrooms, the number of children ranged from 45 to 100 per class; private schools had smaller numbers with a minimum of 45 children per class while government schools had up to 100 children per class. Lessons were dominated by the teacher talking while the children listened or wrote in their books, copying notes from the board. Teachers in the lower primary classes engaged children in chorus songs, making things like beads or art. Interactions in the classroom were minimal; children gave chorus answers, repeating after the teachers or answering when called upon. From these observations, the observers developed evaluation criteria to assess the suitability of the tools in discussion with the research team and teacher representatives. Five key criteria were identified: classroom setting, teaching strategies, cultural relevance, language and structure, and ease of use (Table 2).

**TABLE 2:** Evaluation criteria of the classroom observation checklist, interaction and engagement scale and the teacher-pupil observation tool.

Criteria	CoC	IES	T-POT
Classroom size	The class size did not impact individual child observations	Difficult to note every interaction	Large class sizes complicated accurate observation of interactions using the tool
Teaching strategies	Checklist covered all employed strategies, including common and random teacher-centred learning	Common lecture-based strategies resulted in few observed interactions with the index child	Difficulty noting interaction frequencies because of prevalent teacher-centred learning
Cultural relevance	Items were relevant to the typical Ugandan classroom	Many items not culturally appropriate or easily observable in Ugandan classrooms	Items were not easily observable
Language and structure	Clear and easy to understand	Some items, like determining frequency, were challenging to capture	Letters of representation were confusing, requiring extensive practice and training
Ease of use	Basic training was sufficient, easy for any observer, can be completed by one observer	Extensive training and practice required, multiple observers needed for scoring complexity and lengthy time for administration	Extensive training and practice required, multiple observers needed and more time for effective administration

CoC, classroom observation checklist; T-POT, teacher-pupil observation tool; IES, interaction engagement scale.

### Evaluation findings

#### Classroom setting

Observations spanned 10 schools in the Wakiso district, evenly split between government and private institutions. Each class typically had 1 class teacher, teaching assistants were absent and class sizes varied significantly. Despite challenging pupil-teacher ratios exceeding 30:1 across all classes, it was easier to check off all the items on the CoC as the items were easier to note in a large classroom. The CoC effectively captured critical classroom interactions, such as group work. In contrast, the T-POT and IES encountered limitations because of the nature of the items requiring actual interaction of the child with the teacher or peer within a short observational period. Items on the T-POT and IES required a check for different kinds of interactions between the child with a disability, their peer or classmates and/or teachers. This spanned from compliance, responding to any interaction, initiation of interaction and task behaviour. However, the nature of the classroom and the span of the observation limited the observation of these interactions and when they did occur, were few.

#### Teaching strategies

Table 3 outlines prevalent teaching strategies across observed schools. With a teacher-centric approach being predominant, teaching strategies were uniform throughout the schools observed. The lessons mainly involved the teacher talking and children listening, with interactions occurring almost when the teacher asked questions or assigned a task to the child being observed. The teaching strategies observed across the 10 schools involved children making something, coming to the front, writing (notes from the blackboard), answering questions and singing. Most of the time, children were writing notes off the board in government schools and answering questions in private schools. Children even when involved in what would have been an interactive task like making something (such as beads) were expected to work in silence or engage in chorus responses. The CoC adeptly captured even brief interactions, while the T-POT and IES exhibited limitations, particularly in registering infrequent peer interactions or collaborative tasks.

**TABLE 3:** Teaching strategies observed by school type.

Teaching strategy observed	Government schools (%)	Private schools (%)
Children making something	58.3	41.7
Coming to the front	50.0	50.0
Teacher talking	52.3	47.1
Children writing	63.9	36.1
Children answering questions	46.0	54.0
Songs	59.0	41.0

#### Cultural relevance

Cultural relevance was assessed based on how well the tools’ items applied to Ugandan classrooms, and their observability and usability by Ugandan researchers. Items on the CoC were replaced and the T-POT had an information sheet capturing details of the classroom added to make it more understandable and appropriate for the cultural setting and educational background of the observers. The CoC tool demonstrated effectiveness in capturing items related to inclusion, as a Likert scale checklist, the observers could note the item and had a section to add notes for a deeper comprehension. On the T-POT and IES, the observers struggled to capture cultural nuances and differences in teaching practices within the observed classrooms as these were focussed on time-sensitive incidences of behaviours. They also made the observers feel rushed and limited in their ability to observe the children well because of the emphasis on time rather than the interaction over time.

#### Language and structure

The language and structure of the tools played a significant role in assessing their suitability. For example, the IES used function letters such as I, A, I & A, R, C and A as interaction measures. These were not mainly intuitive, even for experienced observers, who often had to refer back to the definitions document. Mastery of the IES required extensive use to apply it smoothly. Similarly, the T-POT tool had a comparable structure, demanding a high level of familiarity for effective use. In contrast, the CoC tool featured a straightforward checklist with ‘agree or disagree’ questions, making it much easier to follow and complete in a timely manner.

#### Ease of use

Ease of use was considered in terms of training requirements, observer independence and time efficiency. The CoC tool emerged as user-friendly, requiring basic training and enabling reliable observational outcomes within a short time frame. In contrast, the T-POT and IES demanded extensive training, multiple observers, and more time for effective administration, especially in larger classrooms, posing challenges to its applicability in low-resource settings.

In summary, the evaluation criteria and observations highlighted the CoC as the most effective in capturing diverse aspects of classroom dynamics in the Ugandan context, emphasising its user-friendly nature and adaptability to the setting.

Classroom observation checklist reliability was confirmed through a robust Cronbach’s alpha coefficient of 0.80, indicating strong internal consistency, while the T-POT and IES scores could not be calculated because of limited captured interactions, resulting from low interaction frequencies within observed settings.

## Discussion

In this study, the CoC (Collins 2012), T-POT (Martin et al. 2010) and IES (Hunt et al. 1996) were adopted and tested in 10 primary schools in Central Uganda, using the systematic observation theoretical framework to select a classroom observation tool to measure inclusion of children with disabilities. Based on the evaluation criteria of classroom setting, teaching strategies, cultural relevance, language and ease of use, the CoC emerged as the more effective tool for capturing inclusiveness and peer-to-peer support in the classroom. It demonstrated strong internal consistency and was sensitive to the cultural context and classroom dynamics of the Ugandan setting. The CoC was user-friendly and successfully captured the changes in interactions and participation, and was also able to measure peer-to-peer support, which is a key component of the Obuntu bulamu project that this study was conducted within. In contrast, the T-POT and IES struggled to capture nuanced interactions making them less suitable for the Ugandan classroom context, particularly considering factors like classroom size and teacher-pupil ratio. The T-POT’s restricted observation windows and focus on teacher–child interactions hindered its ability to effectively capture peer-to-peer support. The IES did not capture cultural nuances and variations in teaching practices within the Ugandan context. Much as the T-POT and IES were good at capturing active interactions, these were limited and hence reduced the meaningfulness of the findings beyond the observation that there was limited interaction in pairs.

While the CoC was considered most appropriate, it also had some limitations. Specifically, the CoC lacked comprehensive capturing of interaction frequencies and levels within classroom settings, such as the duration and extent of group divisions. The tool did provide comment sections for individual items, allowing observers to note specific occurrences and nuances during observations.

Our study had a small sample size, which limits the generalisability of the findings. Some of the observations did not have two observers but one because of logistical challenges. This might have increased bias and hindered inter-rater reliability that was required for the T-POT and IES. To minimise this risk, we removed outliers from the analysis and guided the observers through feedback and supervision meetings during the study.

Several adaptations and/or translations have been made on standardised tools, measures and devices from HIC to suit the LIC’s settings and increase access to less specialised personnel (Abessa et al. 2016; Montenegro & Valbuena 2009). Tool adaptations allow for proper assessment of the appropriate cultural setting (Gladstone et al. 2010) and can make moves for tailoring interventions to improve inclusion as in our case. The ‘Obuntu bulamu’ study incorporated classroom observations to gauge the participation levels of children with disabilities in mainstream classrooms and measure the impact of the intervention. Selecting a suitable tool that is culturally relevant contributes to the African discourse on promoting knowledge and exploration of African disability and inclusion. Owusu-Ansah and Mji (2013) asserted that it is crucial for African knowledge and methods to be implemented to achieve more meaningful data. The tools from the HIC are a good resource but directly using them for settings like Uganda may not always be applicable, as shown in the testing of the T-POT and IES in this study. With our study, we aimed to not only find an appropriate tool for the ‘Obuntu bulamu’ study but also contribute to the translation, adaptation and evaluation of tools to create relevance and contextualise methods from HIC to suit our setting (Chilisa et al. 2016).

Because of limited interactions observed between teachers and students directly, the IES and T-POT could not adequately provide data on the participation of the children in the class. Within the CoC, we could observe and note changes in participation and involvement over time. As noted by Singal (2008), a child included does not always necessarily translate to a child integrated, hence the need for repeated observations with a tool sensitive enough to observe a shift from a child merely being present in the classroom to a child fully becoming and belonging, which is the ultimate aim of the Obuntu bulamu project.

Martin et al. (2010) have mentioned that classroom observation tools are developed to help address limitations and add to the existing structures. In this study, it was seen that some changes had to be made to some of the existing tools to be able to capture accurate interactions in the study setting. We could in reverse argue that teaching practices need to change in order for us to capture more interactions. However, here too we need to take into account the importance of group response (e.g. chorus) and social and cultural practices which are focussed on the class as a whole rather than the individual child. This is not only because of the teacher-pupil ratio but also to cultural and social values of joint learning and interactions between adults and children and children and their peers. Hence, considering these values in selecting and adapting an observation instrument is key.

### Recommendations

There is a great need for the development and adaptation of culturally relevant and context-specific tools to measure inclusive education in LIC (Nishimura et al. 2009). Development and adaptation of tools can help monitor and support the implementation of inclusive education. With this study, we contributed to testing and selecting a culturally appropriate and context-relevant tool to measure the inclusion of children with disabilities and their peers in the classroom. We recommend researchers test the reliability of the tool in other school settings and further adapt, design and test tools to make meaningful contributions to measuring inclusion in context.

## Conclusion

The study aimed to address the lack of reliable and culturally sensitive tools to assess inclusive practices in LIC like Uganda. Of the adapted and tested tools, the CoC proved to be the most suitable tool for the Ugandan setting, capturing inclusive practices and peer-to-peer support effectively. The findings of this study highlight the importance of considering factors such as class size, teaching methodologies, cultural relevance, linguistic complexity, and ease of use in selecting and adapting classroom observation tools.

By selecting and adapting observation tools that accurately measure disability inclusion relevant to the context, policymakers and educators can gain insights into the effectiveness of inclusive practices and make informed decisions to improve the quality of education for children with disabilities and measure progress towards the United Nations’ goal of ensuring access to quality education for all.
